# Definition of dropout affect rates, predictors and outcomes: evidence from outpatient CBT-E

**DOI:** 10.1186/2050-2974-1-S1-O71

**Published:** 2013-11-06

**Authors:** Louise Pannekoek, Susan Byrne, Anthea Fursland

**Affiliations:** 1 University of Western Australia, Australia; 2 Centre for Clinical Interventions, Australia

## 

There is inconsistency in how we operationalise dropout in clinical research, with studies defining it based on time, number of sessions, therapeutic goals, an identified 'end' of therapy, or the therapist's judgement. The effect of different definitions is poorly understood, but meta-analyses have indicated that they may produce varying rates of dropout and relate to heterogenous client groups. This study aimed to investigate the effect of different definitions of dropout on associated rates, predictors and outcomes in one sample. Analysis involved data from 249 clients (97% female) who received individual outpatient CBT-E. Each participant was classified as a completer or non-completer according to four definitions: before 30 days (10%), before 10 sessions (26%), before prescribed sessions (56%), and non-mutual termination (40%). Analyses indicated that dropout classifications were significantly associated but had poor to moderate agreement. Three definitions were associated with at least one predictor variable and significant variables changed across definitions. Therapeutic outcome was only associated with therapist-judged dropout. Results indicate that inconsistent definitions may prevent accurate interpretation of the dropout literature, and may impede efforts to increase treatment completion. Therapist-judged dropout appeared to be the most useful definition, and we recommend it be the standard definition of dropout.

This abstract was presented in the **Understanding and Treating Eating Pathology** stream of the 2013 ANZAED Conference.

